# Experiences of a nature-based intervention program in a northern natural setting: A longitudinal case study of two women with stress-related illness

**DOI:** 10.1080/17482631.2022.2146857

**Published:** 2022-11-26

**Authors:** Gunilla Johansson, Åsa Engström, Päivi Juuso

**Affiliations:** Division of Nursing and Medical Technology, Department of Health, Education and Technology, Luleå University of Technology, Luleå, Sweden

**Keywords:** Case study, nature-based intervention, natural setting, recovery, stress-related illness, well-being

## Abstract

**Purpose:**

This study explored the experiences of people with stress-related illness participating in a nature-based intervention programme in a northern natural setting.

**Methods:**

A longitudinal case study was conducted with two women participating in a nature-based intervention programme on a farm. Data were collected by semi-structured interviews, diaries, rating scales, and self-assessment. Qualitative data were analysed by qualitative content analysis and quantitative data are presented descriptively.

**Results:**

The theme of finding a source for recovery and well-being permeates all categories. The participants perceived the farm and nature to be a calming refuge; they learned to be in the present and could manage the tasks. In togetherness with each other and the facilitator on the farm they felt understood and confident, experienced joy, and found opportunities for change. They gained knowledge and positive memories and found new approaches in life. Self-assessment questionnaires indicated improvements of functioning in everyday life and reduced stress-related exhaustion at the end of the NBI programme.

**Conclusions:**

Nature-based interventions lasting for a relatively short period seem to promote health and may be a complement to other treatments of stress-related illness. Further research is needed with a larger number of participants and in various natural settings.

## Introduction

Mental illness is a contributor to ill health globally and a considerable cause for people living with disability (James et al., 2018). Stress-related illness is an increasing cause of sick leave in industrialized countries (Eurofound, 2018; OECD, 2012, 2013), and in Sweden it is the fastest-growing cause, especially among women (Swedish Social Insurance Agency, 2016, 2020). Wiegner et al. (2015) found that more than half of those in a working-age population with a primary healthcare appointment for any reason experienced stress to some extent, and approximately half of those reported symptoms of burnout and/or exhaustion.

The diagnosis of exhaustion disorder was accepted by the National Board of Health and Welfare in Sweden in 2005 in order to facilitate more accurate diagnostics and seems to be the most valid clinical equivalent of burnout (Grossi et al., 2015). Exhaustion disorder is caused by long-term exposure to stress with an insufficient opportunity for recovery, is characterized by physical and mental fatigue, and is often combined with symptoms of depression (National Board of Health and Welfare in Sweden, 2003). Physical symptoms, like gastrointestinal problems, headache, dizziness, chest pain, and other bodily pains, are common (Glise et al., 2014), as are sleep disturbances and cognitive difficulties like impaired memory, attention, concentration, and executive function (Grossi et al., 2015).

While the most prevalent risk factors for stress-related illness are a high workload and/or emotional demands at work, stressors in private life can also be a contributing factor (Hasselberg et al., 2014). Work-environment factors, such as low support and the feeling of not having control in one’s job, also increase the risk for stress-related illness (Aronsson et al., 2017). An imbalance between everyday occupations (i.e., employment, domestic work, leisure, rest, recreation, and sleep) is associated with perceived stress and stress-related disorders; for women specifically, an imbalance between doing things for themselves and doing things for others seems to be a common risk factor for stress-related disorders (Håkansson & Ahlborg, 2017).

Cognitive behavioural therapy is an effective treatment that reduces the symptoms of stress and exhaustion disorder (Hofmann et al., 2012; Lindsäter et al., 2018; Salomonsson et al., 2020). A multimodal treatment programme for those with exhaustion disorder conducted by a team consisting of a psychologist, a physician, a physiotherapist, and a rehabilitation coordinator (i.e., an occupational therapist or nurse) has been shown to minimize symptoms of exhaustion disorder, improve the participants’ quality of life, and lessen the amount of sick leave (Van de Leur et al., 2020). Participants in a recovery-focused stress-management programme who were experiencing stress-related health problems reported a positive effect on their recovery behaviours, and they experienced decreased stress and stress-related symptoms (Lisspers et al., 2014).

Spending time in nature offers opportunities for stress relief and has a positive impact on health and well-being (Sempik et al., 2010; Van den Bosch et al., 2018). Even though the health benefits and therapeutic value of nature have been long known (Hartig et al., 2011), the importance of being in nature is often overlooked in modern society (Sempik et al., 2010). In addition, interactions between humans and animals can provide social support, offer psychological and physiological benefits, and improve health and well-being (Fine & Weaver, 2018).

In nature-based interventions (NBI) the goal is to involve people in activities such as gardening, farming, physical activity, or interaction with animals in a supportive, natural environment (Pálsdóttir et al., 2018). NBI can be combined with other treatments such as cognitive behavioural therapy, meditation, and/or counselling (Steigen et al., 2016). There is evidence that NBI has positive effects for people with mental illness (Grahn, 2022). Corazon et al. (2019) reviewed studies with participants with or without illnesses and showed that NBI decreased stress and improved well-being and quality of life. An integrative review (Johansson et al., 2022) concluded that NBI programmes strengthened the health of people with stress-related illness; improved their physical and psychological well-being; and reduced their symptoms of stress. Participating in an NBI also resulted in a reduced need for healthcare and decreased sick leave. A synthesis of qualitative studies conducted by Bergenheim et al. (2021) revealed that people with stress-related illness who participated in NBI experienced positive effects on their health and recovery; they described feeling calm, experiencing joy, finding new insights, and experiencing personal development.

While NBIs are implemented in some southern parts of Scandinavia for people experiencing stress-related illness (Pauli, 2019; Sidenius et al., 2017; Stigmar et al., 2016), such interventions are rare in the Circumpolar North. Reasons can be that the development of NBI has started in the southern parts of Scandinavia, with focus on gardens and gardening (cf Stigsdotter & Grahn, 2003). Implementation of NBI to support health for people, has therefore been easier and in addition there are different policies for healthcare in different regions. The setting for NBI exists in a geographical context that interacts with the client’s experience (Stigsdotter et al., 2011). The natural settings and climate during the seasons are significantly different between northern and southern geographical contexts, which may impact outdoor interventions. Mainly, research of NBI for people with stress-related illness have studied garden interventions (Johansson et al., 2022). Long, cold winters and short summers in northern natural settings may make other NBIs more suitable than garden interventions. The New Nordic Nature Based Service Models (Nordic NaBS) project, of which this study is a part, aimed to develop NBIs suitable for northern conditions. As such, the aim of this study was to explore the experiences of people with stress-related illness participating in an NBI programme in a northern natural setting.

## Methods

### Study design

A longitudinal case study design was conducted in accordance with Yin (2018). This is a suitable design to study phenomenon in real-life settings over a period of time. Multiple sources of data were used because triangulation is desirable in case studies to yield an extensive, in-depth description of the phenomenon under study. A case-study protocol was created to plan, describe, and guide the data-collection procedure (Yin, 2018), and a computer-based case study database was created to gather and organize the data collected. According to Yin (2018) an important principle in case studies is to maintain a chain of evidence, which means explicit links between research question, collected data and conclusions. In this case study it is strengthened by citations from interviews and diaries. Data were collected through semi-structured interviews, diary entries, and rating- and self-assessment scales.

The first author was present during the second and fifth session of the NBI programme to observe the environment and the participants’ activities and interactions. The observations were done with limited participation so as to not interfere in the participants’ interactions with the facilitator and with each other (Holloway & Galvin, 2017). The observations were documented in memos after each session in accordance with the observation protocol created for this study; while these notes were not analysed, they were consulted when preparing interview guides for the individual semi-structured interviews.

### Study setting—the NBI program

The NBI programme under investigation was conducted on a small farm located 7 km outside a city in northern Sweden; the participants attended this five-session programme once a week in the evening between late winter and early spring, and each session lasted for two-and-a-half hours. The facilitator had a background as a conversational therapist in social services and was qualified in motivational interviewing, equine-assisted social work, and as a forest bathing guide. When the programme began the ground was covered with snow except for paths and roads, but most of the snow melted by the final week of the programme. There was a horse stable and a paddock on the farm, and next to the paddock there was a fireplace covered with a roof and benches on which to sit. Animals on the farm included two horses, one dog, and a chicken coop with four hens and a rooster. The farm was surrounded by a forest.

Most of the activities took place outdoors, but some were conducted in the stable. Each session started by the fireplace, with the facilitator having a follow-up from the previous session and presenting her plan for that evening’s session. The activities included feeding, brushing, petting, and practicing leadership with the horses, cleaning the horse stalls and the chicken coop, packing hay, and “forest bathing” (i.e., laying on the ground in the forest, using the senses, and relaxing). At the end of each session everyone gathered around the fire and reflected on their experiences from the evening’s session.

### Procedure

In this study the NBI programme on the farm could include a maximum of four participants. The inclusion criteria were people 18 years of age or older who were experiencing stress-related illness. Participants were recruited with a purposive sampling during the winter 2021. The head of a primary healthcare centre was contacted and granted permission to inform patients who met the inclusion criteria about the study. Two conversational therapists on the primary healthcare centre working with patients with stress-related illness gave patients who met the inclusion criteria a short-written information about the study and the contact information for the first author. Three women contacted the first author by telephone and received oral information about the aim and procedure of the study. All of them expressed interest to participate and received a more comprehensive written information and a consent form by post which the signed and sent back. One, however, withdrew her participation after the first session.

### Participants

Two women about 55 years old chose to participate. One lived with her partner and two children, and the other lived alone with her dog; one had a high school education and the other had vocational training; one worked in the manufacturing industry and the other worked in social care, and both were permanent full-time employees. Just over a year before the study began both women had been on full-time sick leave due to exhaustion disorder and had gradually increased the amount of time they worked until their health insurance ran out. While one of the women chose only 85% employment due to her stress-related health issues during the period of this study, the other woman engaged in tasks at work that differed from her usual responsibilities due to her stress-related health issues. They both had attended cognitive behavioural therapy. While one of the women had participated in an NBI programme on the same farm one year earlier, the other had no previous experience with NBI.

### Data collection

Multiple sources of data were collected in this study to reach both in-depth and broad descriptions of the phenomenon under study via data triangulation (Yin, 2018). The different data sources are described below.

*Semi-structured interviews* were conducted with each participant a total of four times: before the NBI, after the second or third NBI session, approximately one week after the last NBI session, and five to six months after the NBI programme concluded. A separate interview guide (Kvale & Brinkmann, 2014) was formulated for each interview occasion. The initial interviews were based on the participants’ experiences spending time in nature and their expectations of the NBI programme. The interviews during and shortly after the NBI programme were based on the participants’ experiences during the NBI sessions and details from their earlier interviews and the observations. And the interviews five to six months after the NBI programme were based on the participants’ thoughts and experiences in the period following the NBI programme. The interviews lasted from 25 to 70 minutes, and all were conducted via Zoom or over the telephone due to the COVID-19 pandemic; they were recorded and then transcribed verbatim. The participants were in their homes during interviews, which, in accordance with Holloway and Galvin (2017), may have helped the participants feel more relaxed, resulting in richer data.

*Diary*. Two weeks before the first NBI session the participants received a diary in which they were asked to reflect about their experiences and thoughts of the NBI programme and other nature-related activities. According to Holloway and Galvin (2017), diaries reflect a person’s insider view of their experiences and life. Rating scales, one for stress and another for health, were attached to the diary. The rating scales consisted of a 10 cm-long visual analogue scale (Polit & Beck, 2021) for each area that ranged from 0 (low) to 10 (high). The participants were asked to rate their experiences of stress and health levels on a daily basis from two weeks before the start of the NBI programme until the end of the NBI programme.

*Questionnaires*. Two self-assessment questionnaires—the outcome rating scale (ORS) to measure functioning in everyday life and the Shirom-Melamed Burnout Questionnaire (SMBQ) to measure stress-related exhaustion—were administered three times: before the NBI, at the end of the programme, and at a long-term follow-up five to six months after the end of the NBI programme. The ORS is a questionnaire that covers four areas of functioning: individually (personal well-being); interpersonally (family, close relationships); socially (work, school, friendships); and overall (general sense of well-being). The questionnaire consists of one 10 cm-long visual analogue scale in each area that ranges from 0 (low) to 10 (high). The participants rated their functioning during the previous week (Andersson & Marklund; Miller et al., 2003). The SMBQ was designed to estimate level of burnout/stress-related exhaustion and to evaluate treatment effects. The questionnaire consists of 22 items rated from 1 (almost never) to 7 (almost always) and includes four subscales: emotional and physical fatigue, listlessness, tension, and cognitive weariness (Lundgren-Nilsson et al., 2012).

### Data analysis

Triangulation was conducted, meaning that we used multiple sources of evidence by means of multiple data sources (data triangulation) and different methods (methodological triangulation; Yin, 2018). The repeated interviews and daily diary entries generated data with rich descriptions of the participants’ experiences. Data from the self-assessment ratings corroborated these experiences, showing both similar patterns and variation (Polit & Beck, 2021; Yin, 2018), which was assessed as sufficient for analysis. Data was analysed with suitable methods for each source of data.

Qualitative content analysis, in accordance with Lindgren et al. (2020), was used to analyse the interview transcripts and reflections from the diaries. Data analysis was performed separately depending on when data were collected: the interview texts and diary notes from before the NBI programme; the interviews conducted during and shortly after NBI and the diary notes after the start of NBI; and then interview texts from the long-term follow-up interviews. This resulted in three separate analyses. For each analysis the data was first read in whole to obtain an overall sense of the content. Then meaning units, parts of the text that answer the research question, were extracted and condensed; this means the text was shortened while retaining the core of the content. The next step was to categorize the condensed meaning units to bring them together based on similarities and differences in content, which was done in several steps. Finally, the categories from all three analyses were compared and interpreted to identify a theme that described the underlying meaning in the text (Lindgren et al., 2020).

The rating scales from the diaries and data from the questionnaires were analysed and presented descriptively. *Outcome rating scale (ORS*): The score on all four scales were added to a total score (range: 0–40). Values over 25 are considered to indicate good function in everyday life (Andersson & Marklund; the National Board of Health and Welfare in Sweden, 2021). Miller et al. (2003) found that ORS has adequate validity, solid reliability, and high feasibility. *The Shirom—Melamed Burnout Questionnaire*: The result was calculated as the mean of all items, and higher scores indicate a worse condition. In this study, the four items measuring tension were excluded in analysis in accordance with the Lundgren-Nilsson et al. (2012) study of the construct validity of SMBQ. A cut-off for SMBQ at 4.4 is suggested, indicating stress-related exhaustion for scores at or above the cut-off (Lundgren-Nilsson et al., 2012).

### Ethical considerations

This study was approved by the Swedish Ethical Review Authority (Dnr 2020–00759). The participants received oral and written information about the aim and procedure thereof and were informed their participation was voluntary and they could withdraw at any time without specifying any reason. Written consent was obtained from all participants and all data were handled in a confidential manner. Because of the participants stress-related illness simple questionnaires that did not require a lot of effort to fill in were chosen. They were informed that daily notes in the diaries was not mandatory. The interviewer, who is a nurse, paid attention to the participants reactions during interviews and the participants had her contact information so they could get in touch at any time if needed.

## Results

The analysis of the interviews and diaries resulted in an overall theme and a total of eight categories. The categories are presented separately for before NBI, during and shortly after NBI, and long-term follow-up after NBI (see, Table I). The categories are supplemented with quotations from the interview texts and diaries. The results from the analysis of the rating scales and questionnaires are presented descriptively. The names in quotations and figures are pseudonyms.

**Table I.** Overview of theme and categories.

Theme	Categories
	***Before the NBI***
	*Spending time outdoors enjoying nature*
	*Looking forward to participating in NBI*
	***During and shortly after the NBI***
*Finding a source for recovery and well-being*	*Finding a calming refuge*
	*Learning to be in the present and managing tasks*
	*Feeling understood and confident*
	*Experiencing joy and finding opportunities for change*
	***Long-term follow-up after NBI***
	*Receiving positive memories and knowledge*
	*Finding new approaches in life*

### Finding a source for recovery and well-being

The NBI enhanced the participants’ ability to enjoy their contact with nature. Nature was found to be a place for recovery, a calming refuge. To become more aware of their sensory impressions in nature and togetherness with the animals enabled them to be in the present. Meaningful and enjoyable activities in peace and quiet encouraged them to slow down and to succeed with tasks further supported their personal development. Confidence in each other and in the facilitator strengthened their feeling of being understood and valued. The joy and happiness they experienced throughout the programme enhanced their sense of well-being and they learned, practiced, and began to prioritize their recovery.

### Before the NBI

#### Spending time outdoors enjoying nature

One of the participants took daily walks with her dog in nature near her home; the other participant walked almost every day. On their days off, they both sometimes took trips into nature that was further from home.

*I have to go out and make sure he [dog] is well. I don´t know if I would have gone out and walked by myself otherwise, but now he is the most important. (Elisabeth, interview)*

The nature awakened their senses and led to a restful experience that made it easier to be in the present. They expressed feeling joy and being imbued with strength and energy. One participant appreciated the white snow, cold weather, and fresh air, even though the cold sometimes felt unpleasant.

*I feel that it is kind of restful to be in nature. I put my mobile away, it´s like I´m here and now. (Elisabeth, interview)*

*It was nice to walk and enjoy the sun, fresh air, bird twitters, the sound of water dripping from a roof. I enjoyed it and I felt joy. I became alert. (Maria, diary)*

#### Looking forward to participating in NBI

Both participants looked forward to participating in the NBI programme. The one who had participated earlier in NBI had positive experiences and a desire to be on the farm again. They expected a calm environment and calm outdoor activities and wished to spend time with the horses. There was some tension to meet the other participants; they expressed feelings of being reserved around people they did not know, but they also hoped to meet others with similar experiences who would understand their situation.

*At the same time exciting and nervous to meet others … because you feel a little exposed, but it is worth it. (Maria, interview)*

#### Self-assessed functioning in everyday life and stress-related exhaustion

Before the start of the NBI programme the scores of functioning in everyday life (ORS) were lower than what is considered to indicate good functioning in everyday life (see, Table II). Scores of stress-related exhaustion (SMBQ) indicated stress-related exhaustion for both participants before the start of NBI (see, Table II).

**Table II.** Participant scores of functioning in everyday life (ORS) and stress-related exhaustion (SMBQ) before, after, and at long-term follow-up after NBI. (The names are pseudonyms.)

	Before NBI	After NBI	Long-term follow-up after NBI (5–6 months)
*Outcome rating scale (ORS)*			
Elisabeth	18	25	20
Maria	22	28	13
*Shirom-Melamed Burnout Questionnaire (SMBQ)*			
Elisabeth	5,2	4,4	3,8
Maria	4,6	3,2	4,9

ORS-values over 25 are considered to indicate good functioning in everyday life. SMBQ-scores at or above 4,4 indicate stress-related exhaustion.

### During and shortly after the NBI

#### Finding a calming refuge

Attending the NBI-programme was described as a positive experience, which was in accordance with the participants expectations. They felt good to go to the countryside but still not far from the city. The small farm was described as a nice and calm environment and one of them expressed that a large farm would have been overwhelming for her. They described their experiences during the sessions as pleasant and looked forward to the next session.

*I become calm from being in that environment, I feel good being with animals and in nature. It´s like I don´t feel stressed in any way, but I´m calm and harmonious there. (Elisabeth, interview)*

They appreciated contact with the animals, especially the horses, but neither expressed a desire to ride them. None of the participants had prior experience with horses and they had respect for them because of their size, but they trusted the horses and felt calm and harmonious when around them. Being close to the horses led to feelings of affinity and they were emotionally affected. The participants also appreciated watching the dog run around the farm and the hens walking around the yard and talked with them when they came close. One of the participants reported mild allergy symptoms, but she did not consider this to be a problem and simply avoided going into the hen coop.

*I feel happy in some way. Being with the horses gives me a good feeling. I have not been afraid of them at all, but I respect them because they are big, but still I trusted the horses. I only have a positive feeling about it. (Elisabeth, interview)*

*It is something special, standing next to the horse, taking care of it, and noticing that it likes it and seeks contact with me. I am calmed by the horse being calm. I was emotionally touched. Even though we do not know each other, it [the horse] let me be there. (Maria, diary)*

During forest bathing, when the participants laid down in the forest, their senses were stimulated, and they enjoyed the experience. They appreciated the silence but admitted that traffic sounds from the nearby road disturbed their experience. The animals also stimulated their senses, such as the sound of the horses chewing hay and the cackling of the hens. They enjoyed the crackling of the fire during the gatherings around the fireplace. One participant explained that she learned how to use her senses again and became more aware of sensory impressions of nature. She recognized a feeling she had experienced as a child and felt that she previously had not understood the importance of spending time in nature. Both participants liked being outdoors on the farm, and they described the experience as fresh and nice, even though it was sometimes cold and sloppy, and muddy when the snow was melting. They were wearing clothes according to the weather to avoid freezing.

*Breathing the cold natural air. The scent of the forest. Bird twitters. Trees cracking. Looking at the treetops, the sky, the clouds. Catching a glimpse of the sun. Feeling the warmth of the reindeer skin beneath me. I removed my gloves and touched the ground. Lingonberry sticks, twigs, trees. (Maria, diary)*

*It´s like when I was a child and was outdoors and relaxed. I haven´t understood how important it was. So, I should have continued with it more. (Maria, interview)*

#### Learning to be in the present and managing tasks

The calm activities could be adapted to suit the abilities of the participants and they appreciated that there were not too many activities on the farm. They did not feel pressure to perform; they only did what they were able to handle. They did one thing at a time at their own pace, took breaks, and completed their tasks in peace and quiet. The participants experienced being in the present and enjoying the moment, partially influenced by the animals, which they perceived as also being in the present. One participant expressed that the time spent on the farm seemed to take longer than usual in a positive way, and she believed it was because they were encouraged to focus on what they were doing, not on the time.

*I feel no stress or pressure to do anything. If I feel that it [some activity] don´t feel good to me, I can discontinue if I want to. There are no expectations on us, it´s entirely up to us. (Elisabeth, interview)*

*When I´m there [on the farm] it is like I´m there and nothing else exists. The time feels so long. It feels like I´m only doing what I´m doing at the moment. It´s a nice experience to feel that I enjoy that moment. (Maria, interview)*

The participants described forest bathing as nice and enjoyable. They both felt they needed to learn and practice how to relax. It took them a while to calm down and find peace, so they needed enough time. It was difficult to relax if they were too cold, but they were warm and cosy when they lay on reindeer skins covered with sleeping bags. One participant experienced strong feelings similar to deep meditation during her forest bath, which touched her deeply and she sometimes nearly fell asleep.

It takes a while before i really relax, so i need to have enough time. (Elisabeth, interview)

Taking care of and feeding the animals made the participants feel important. They felt good and that they stretched their limits when they were able to handle the horses in spite of not having any experiences with equines. The horses were obedient, which made it easier to succeed. One participant described that she once during forest bath began to think about that she is afraid of bears, but she overcame her fear and remained laying. The experience of coping with challenging tasks was described as strengthening the participants.

*It feels like a strength when you actually get the horse to do what you want. Although you feel that you know nothing about horses … it was so fun. (Maria, interview)*

*Of course, I´m afraid of bears, and thought: What do I do if a bear comes? But then I decided: I don´t care! It was a cool feeling to just continue to enjoy what I´m doing. (Maria, diary)*

#### Feeling understood and confident

Meeting the other participant in the NBI was described as less stressful than meeting people at work. The participants felt as if they were in the same situation and therefore understood one another; it was easy to talk with someone with whom they could share their experiences. It meant a lot to know they were not the only person with stress-related health issues and meeting each other gave them hope. One participant asserted she would not have managed to meet the other participants when she was severely ill and more fragile due to her exhaustion disorder than she was at the time of the NBI programme.

*It´s easier to talk with someone who knows than to explain to someone who has not been through it [stress-related illness]. It´s probably hard for people who have not been there themselves to understand how it feels. (Elisabeth, interview)*

*It actually feels good to know that the other [participant] know that I have it [stress-related illness] too. It´s nothing, now we are two about it. So even if it is different, I have no idea about the others all the more, but there is some similarity. (Maria, interview)*

The participants appreciated gathering around the fire, which facilitated a nice, warm, and calming atmosphere for conversations. They drank tea or coffee and discussed their experiences from the session. The participants inspired and supported each other, but neither shared their thoughts or asked questions that could be considered too private. The participant who lived alone especially liked the social contact during the NBI.

*We talked about different things … It felt good, not difficult. If you don’t feel like talking so much, you don’t have to. It depends on how you feel from time to time. (Elisabeth, interview)*

*But you are careful, it is so private … If you have an illness or something like that, you may feel … I don´t want to ask them things that they might not feel like answering on. (Maria, interview)*

The participants perceived the facilitator was knowledgeable about nature and animals and showed consideration for the animals. She was described as pedagogic when she guided the participants during activities, and they knew they could ask her questions anytime they felt uncertain about something. The participants perceived the facilitator as calm, supportive, and down-to-earth and expressed that they felt comfortable in her presence. They felt that she understood them and was aware of their needs. As the facilitator maintained control during activities, the participants were able to relax and trust that she was there and would take over if something happened, which increased their confidence.

I trusted that she would never let us be with a horse that was not calm. (Elisabeth, interview)

*When I was working with the horse last time … in one of the exercises I just said: I want you [the facilitator] to be next to me, and then she came and stood next to me. (Maria, interview)*

#### Experiencing joy and finding opportunities for change

Both participants experienced that attending the NBI awakened feelings of joy and increased their energy. They had fun and laughed when they were on the farm, and these feelings of happiness and satisfaction continued after the sessions. They both admitted to feeling tired after the sessions, but it was a positive tiredness, not exhaustion. One participant explained that she slept better after she had been on the farm; the other stated it could be difficult to fall asleep because she was so exhilarated with all her impressions.

What I have done at the farm gives me energy. Joy. Feels so good. (Maria, diary)

*I get tired of being outdoors, but at the same time I´m filled with energy. Being tired because of an activity can be positive. I´m not tired in a negative way, but I´m tired and able to sleep better. (Elisabeth, interview)*

The participants learned more about recovery and how to handle stress by being in nature, and they expressed a desire to use these experiences in their everyday lives. They thought about being outdoors in nature more and considered what they would be able to do on their own after the programme concluded. One participant expressed that the NBI started a process and opened her mind to creative thoughts on her development in life.

*Maybe I should do that more, go out into the forest and lie down. It´s not so stupid to lie down under a tree. (Elisabeth, interview)*

*I think more about what I can do better for myself in my life. What changes can I make, do I want to make? How should I change my life so I can feel good? (Maria, diary)*

Both participants tried to spend more time in nature, and they enjoyed these experiences more than they had before participating in the NBI. When taking the same walks they had before the programme, they described the activity as a way to unwind and recover. One participant sometimes sat or lay down on a reindeer skin in the forest, and the other considered going into the forest to meditate. One of the participants spent time in her greenhouse caring for her plants, or she would simply sit and drink tea; she recognized the feelings she experienced on the farm when she was gardening, and this caused her to feel calm and in the present. They were inspired to begin engaging in new activities such as gardening or to be involved in the care of horses, but one of them was worried that it would be too demanding on her health.

*I feel good when I spend time in nature, so I want to do it … I think more about being in nature … and thus to enjoying it too. I have laid down outside and got some fresh air … it is great. (Maria interview)*

*Maybe I should try to get in contact with … [horses]. I may not need to ride, but to be with a horse, to be there and pet them and just take care of … [them]. (Elisabeth, interview)*

Health issues such as pain and tiredness sometimes hindered the participants from spending time in nature. One participant went to the NBI sessions despite feeling pain, which made her feel good. While both participants spent time outdoors in cold weather, bad weather sometimes made them to stay indoors. The participants did not experience any disadvantages associated with attending the NBI, and they both expressed they wished the programme was longer.

*It was hard to lie in the forest once [because of pain], but … I would not want to be without it either. The positive benefits are more … than what felt negative. (Maria, interview)*

#### Self-assessed functioning in everyday life and stress-related exhaustion

At the end of the NBI programme ORS indicated good functioning in everyday life for both participants. SMBQ were at the cut-off for indicating stress-related exhaustion for Elisabeth and not indicating stress-related exhaustion for Maria. ORS showed improvement of functioning in everyday life and SMBQ showed reduced stress-related exhaustion at the end of the NBI programme for both participants (see, Table II).

### Long-term follow-up after NBI

#### Receiving positive memories and knowledge

Overall, both participants viewed the NBI programme as a positive experience and explained that remembering their experiences in the programme resulted in positive feelings. One participant admitted to initially being sceptical of the forest bathing, but she grew to like this activity. Both participants expressed that meeting another person in a similar situation to theirs and exchange experiences was helpful, though one of them thought that it was challenging for her to open up and show her weakness. One participant asserted it would have been beneficial if she had attended the NBI earlier, when her exhaustion disorder was worse. They both agreed more people should have the opportunity to participate in an NBI.

*Very positive, it really is. I absolutely would not want to be without it [the NBI-program]. It shows a way how you can go … (Maria, interview)*

*I felt a little sceptical about the forest bathing. It actually sounds quite fuzzy before you’ve tried it and understand … then it is positive. (Elisabeth, interview)*

Both participants had learned and considered the use of nature to reduce their stress and improve their well-being more often after the NBI programme. One participant used what she learned during the NBI to reduce her stress while at work; the other had a greater interest in nature afterwards and considered being in nature as an alternative way to achieve and maintain balance in her life that would allow her to avoid exhaustion. One of them did not care about the weather as long as she was not too cold; she liked the winter and snow because it made everything brighter outdoors. The other participant thought that bad weather hindered her from going outdoors.

*I have recovered a lot. I have gone aside and meditated or taken a walk. I have felt what I need to do. (Maria, interview)*

#### Finding new approaches in life

Neither of the participants started engaging in new activities after the NBI programme. While one participant spent more time in nature than she had before the NBI, the other did not. When the participants spent time in nature, they took time to receive sensory impressions more than they did before the programme. Being in nature felt different and was more restful, and they enjoyed these experiences to a greater extent than they did before participating in the NBI programme.

I stop for a moment, are more attentive and breathe. It calms down the stress. (Elisabeth, interview)

*I hear a lot of birds. I do not know if there are more birds now or if I hear them better now. (Maria, interview)*

Both participants had learned to “just be” and to appreciate what was happening at any given moment. One of the participants felt she was in the present, able to focus on what she was doing and enjoy the little things in her everyday life to a greater extent. She took her time and did not rush through activities like she had before the programme. She “listened inward” more closely to what she needed, wanted, and was able to manage. One of them began to set limits for her work and prioritized her leisure time and recovery; the other participant reflected on what she considered to be important in her life and what was not and focused on her recuperation to a greater extent than she had before. One of the participants expressed that even though some of the activities made her feel tired they also filled her with energy.

*I see what is happening right now and capture the moment. It feels like time is standing still, not rushing on. It feels good. A lot has changed. (Maria, interview)*

*When I´m at work, I´m doing my work and when I leave, I try to leave it. I feel that it is more important for me with recovery. (Elisabeth, interview)*

One of the participants felt good at the time of the long-term follow-up interview: she believed the NBI programme helped her, but she also stated that time may also have played a role in her recovery. The other participant expressed that she had struggled with her stress-related illness and it took a long time to recover; she still struggled.

*I think I feel pretty good anyway. I can manage my job, and I feel less stressed when at work. But I don´t know if it was time that did it, or if I gained something., I think you get it from all the things you do and experience. (Elisabeth, interview)*

*It has been a struggle for me … and there have been setbacks. It takes a long time to move forward. (Maria, interview)*

#### Self-assessed functioning in everyday life and stress-related exhaustion

At the time of long-term follow-up, scores of ORS was again, as before the NBI programme, lower than what is considered to indicate good functioning in everyday life for both participants. SMBQ was not indicating stress-related exhaustion for Elisabeth. SMBQ for Maria was again indicating stress-related exhaustion (see, Table II). By the time of the long-term follow-up, Maria was on part-time sick leave, which she acknowledged may have affected her long-term follow up scores.

ORS-values over 25 are considered to indicate good functioning in everyday life. SMBQ-scores at or above 4,4 indicate stress-related exhaustion.

**Figure 1.**
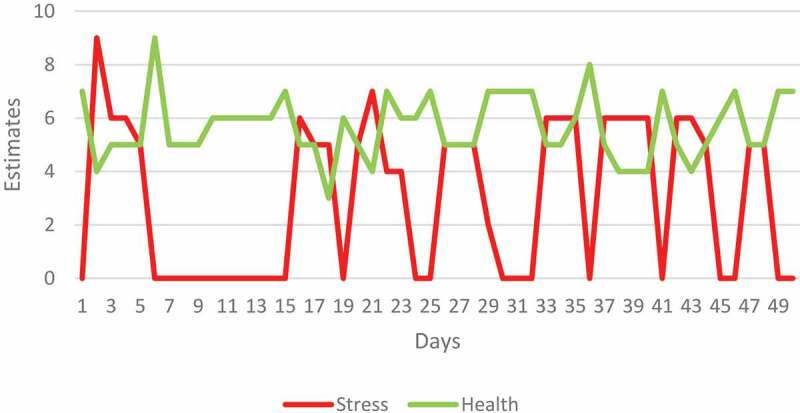
Daily estimates of stress and health recorded by Elisabeth in her diary. The NBI program began on day 15.

**Figure 2.**
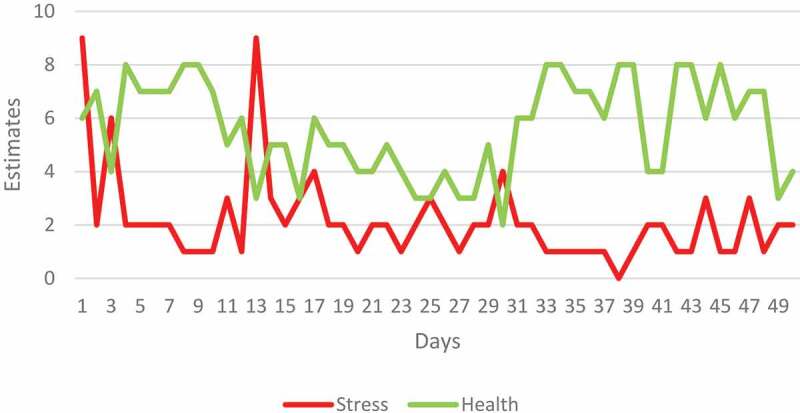
Daily estimates of stress and health recorded by Maria in her diary. The NBI program began on day 15.

The daily stress and health estimates the participants recorded in their diaries did not demonstrate any obvious trends (see, Figures 1 and 2).

Elisabeth was on sick leave from day 7 until day 15 due to a mild injury, which may explain her low estimates of stress during that period.

## Discussion

In this case study we aimed to explore the experiences of people with stress-related illness participating in a NBI programme in a northern natural setting. The results reveal the participants found a source for recovery and well-being. They perceived the farm and nature to be a calming refuge, they learned to be in the present and could manage the tasks. In being together and with the facilitator on the farm the participants felt understood and they experienced confidence and joy, finding opportunities for change. They gained knowledge and positive memories and found new approaches in life. Self-assessment questionnaires indicated improvements of functioning in everyday life and reduced stress-related exhaustion at the end of the NBI programme. The case study showed that on the farm and in nature the participants found a source for recovery and well-being. According to Hörberg et al. (2020), people living with stress-related illness experience well-being in situations where they feel an unconditional beingness, which means not having demands on oneself and not having to perform. Supportive environments, like nature, where they can be in peace and quiet and be able to relax, can support such feelings and promote well-being in everyday life. In addition, animals can bring forth feelings of well-being through their affection and companionship. Interaction with animals can be less demanding than being with other people, even if the animal needs care (Hörberg et al., 2020). According to our results, spending time with the horses was highly appreciated and affected the participants emotionally in a positive way.

This study’s results show that participants looked forward to taking part in the NBI programme and they expected the setting and the activities to be calm. Edvardsson et al. (2005) found that people are sensing an atmosphere of ease in supportive care environments, a feeling that is promoted if the environment exceeds their expectations. Supportive care environments promote experiences of being relaxed and safe and being in a calm environment enable a person to follow their own rhythm. The setting can give the possibility of social relations and sharing of experiences which contribute to a sense of ease. A care environment support experiences of safety if it meets a person´s needs and the staff are confident in their care. In our study, the results show that the participants expectations were fulfilled, and the setting met the aspects of a supportive care setting in accordance with Edvardsson et al. (2005).

In our study, results show that being on the farm and in nature were described as a calming refuge. The participants did one thing at a time and appreciated being in the present. This can be understood from Kaplan and Kaplan’s attention restoration theory (ART), which emphasizes the importance of spontaneous attention to reduce the attentional fatigue of directed attention used when concentration is needed, which requires energy. Spontaneous attention is used in environments with moderate stimulation, such as nature, and it is restful and promote recovery. This occurs when something spontaneous catches and holds one’s interest without effort, such as sensory impressions in nature, but also when one performs undemanding activities. Spontaneous attention is also about distancing oneself from stressful thoughts in everyday life and provides opportunities to reflect on life (Kaplan, 1995). Our study results show the participants felt that time slowed down, and that they could reflect on changes in their lives. Additionally, the results from the self-assessment scores confirm a decrease in stress-related exhaustion during the NBI.

Participants in our study experienced activities such as forest bathing, during which they laid down in the forest—alone but not lonely—as safe. This can be understood from the Supportive Environment Theory (SET), which combines social engagement, well-being aspects, and environmental aspects to explain how natural environments in the context of NBI are supportive for people with stress-related illness. People with a low sense of well-being tend to have a greater need for a supportive, safe, and stable environment with limited stimulation, like nature. Because of decreased capacity for social engagement, they may need the opportunity to be alone in nature. NBI offers challenges on different levels from passive experiences in nature to more active engagement that is required for example, in activities with horses. SET emphasizes the need for people with stress-related illness to choose settings, activities and interactions with others on their own terms according to current needs and capabilities (Bengtsson & Grahn, 2014; Grahn et al., 2010). The participants in our study appreciated that it was no demands or expectations on them in activities and interactions with each other. Alsén et al. (2020) showed that when affected by stress-related illness, the person might lose previous functions, which contribute to difficulties in managing everyday life. Every task is described to be a challenge, leading to loss of control and loss of self-recognition, why realistic and achievable goals are important for their recovery. Tasks in NBI, which can be adapted to suit the participants current needs and capabilities can promote experiences of control.

The participants in the case study experienced that during NBI they were in the present. They did one activity at a time and enjoyed the moment. Eriksson et al. (2011) found that when participants in a therapeutic gardening programme engaged in activities, they became absorbed in the present. They experienced happiness and joy and became more reflective and sensitive to their own thoughts. Symbolic thoughts awakened about the parallels of processes in nature and in their lives. The participants in our study started to reflect about possibilities for changes and new approaches in everyday life.

Participants in the case study appreciated to “just be” in the moment. Hammell (2004) describe *being* as essential and central in life. Being can be defined as time to enjoy the moment, appreciate nature for instance, be mediative, reflect and discover ones self. Voluntary and self-chosen activities for pure enjoyment and pleasure offer opportunities of being and may consequently be meaningful for people with illness to reflect on their life.

Eriksson et al. (2011) found that a therapeutic gardening programme inspired participants with stress-related illness to engage in enjoyable activities in everyday life, which contributed to balance in their lives. They received more energy and happiness when they continued with activities that were meaningful for them. The participants in our case study were inspired to start with new activities in connection with nature and animals; however, neither of them realized these plans during the time of the study. Based on SET (Bengtsson & Grahn, 2014), it can be understood that the participants still needed time in a supportive and safe environment in order to become more emotionally involved and foster their own active search for health promoting activities in nature.

Even if the participants in the case study did not introduce new activities in their everyday life, they expressed that being in nature felt different after the NBI programme. Nature was perceived as more restful, and they enjoyed it more than before. Grahn et al. (2021) emphasize the experience of calm and connection in nature, which reduce stress and promote personal development. In nature there is change and movement that shows life, while at the same time there is stability and peacefulness. The hormone oxytocin is released by positive experiences in natural settings, increasing trust and well-being and promoting a sense of belonging and an affinity to nature. To be able to experience this, relations with nature should take place when people are alone and in silence.

The participants expressed that they stretched their limits when they succeeded in handling the horses and felt important when they took care of and fed the animals. According to Zulkosky (2009), self-efficacy is about the perception of being able to manage specific situations; it influences peoples’ motivation, choice of activities, experiences of stress, and emotional reactions. To enhance a persons’ control over their life, self-efficacy can be strengthened by experiences of being successful with tasks. In treatment situations, activities can be adapted to avoid failure, such as in our study when the horses were obedient and easy to handle it was easier for the participants to succeed. To watch others—such as other participants in NBI—manage a task can also strengthen self-efficacy (Zulkosky, 2009). Individualized activities in NBI that suit the participant’s abilities can offer opportunities to succeed with a task and strengthen the person’s self-efficacy.

In our study, the participants trusted the facilitator as her support made them feel confident. Wästberg et al. (2021) emphazise the facilitators important role in NBI to create a safe atmosphere, see each participants needs and adapt the activities to challenge them to promote personal development. According to Palsdottir et al. (2014), a permissive atmosphere in NBI supported by the staff is important to make people with stress-related illness feel relaxed and secure. Being amongst others with similar experiences allows them to feel mutual understanding, as equals, and promotes their opportunity to be themself, like the participants in our study appreciated meeting another person in a similar situation and being able to share their experiences of stress-related illness.

The participants in our case study received new knowledge and found new approaches in life. The self-assessment scores showing an increase of functions in everyday life during the programme. According to Orem, nursing should support people to promote their own health by strengthening their capacity of self-care. One universal self-care need identified by Orem is to maintain balance between activity and rest (Orem, 1995), which is very important for people with stress-related illness. NBI can strengthen the self-care ability of people with stress-related illness so they can find and maintain balance in life and improve their health. The improvement of functions in everyday life during the programme did not maintain until the long-term follow-up, which may depend on the short duration of the NBI programme. Grahn et al. (2017) found that longer NBI programmes resulted in better outcomes than shorter NBI programmes.

Geographical regions have a biological diversity of flora, fauna, and climate that are distinct from each other (Seeland, 2011). The northern natural environment is of specific importance for the people living there, despite the often long periods of cold weather. Cunsolo Willox et al. (2012) showed that people’s sense of place increases natures’ health promoting effects as the familiar environment can be stress reducing and refreshing. In our study results, the participants’ experience of the weather sometimes decreased their possibility to relax. Bielinis et al. (2018) studied peoples’ responses to 15 minutes of forest bathing in wintertime and showed that exposure to a forest environment during winter evoked psychological relaxation. So, NBI during wintertime in environments where temperature is low is indicated, and shorter interventions might be enough for stress reduction.

### Methodological considerations

This study has limitations, such as the fact that the NBI programme was of short duration, which may have limited the outcome. Using a case study protocol to guide the data collection process, as well as the use of data from multiple sources, strengthened our study as data triangulation was done (Nilmanat & Kurniawan, 2021). The ratings in the diaries and self-assessment questionnaires contributed to the participants’ estimates and showed possible changes during the period. Altogether, the collected data were assessed to be sufficient to answer the aim of the study. The use of a computer-based case study database to organize the data collected and efforts to maintain a chain of evidence can be seen strengthening the trustworthiness of the study.

Following the case over time with repeated interviews and questionnaires is a strength (Nilmanat & Kurniawan, 2021). The first author’s participation during some of the programme sessions was helpful to understand the context and supported the interviews. However, this may have affected the participants’ experiences. During the long-term follow-up one of the participants experienced health problems that may have affected that part of the result. The data, analysis, and results were discussed among the authors to strengthen the trustworthiness of the study.

The NBI programme in this study, could include a maximum of four participants and all who contacted the first author were included in the study, but one chose to withdraw after the first session. The participants in our case study were females and about the same age, which means that we had a homogeneous sample. As stress-related illness is more common among females (Swedish Social Insurance Agency, 2020), the purposive sampling included the ones who gave their consent to participate. No special actions were taken to recruit males. A heterogeneous sample with a variation in gender and age could have resulted in more variations of experiences (Holloway & Galvin, 2017). One participant had previous experience of NBI which probably affected her expectations and experiences.

Lack of generalizability because of inclusion of few participants is one of the most quoted weaknesses of case studies. The number of participants is however, not of great importance. Instead, it is important that the selection of participants is strategic and appropriate to be able to answer the research question (Flyvbjerg, 2006). Our case study included two participants who we considered to be representative for the population we aimed to study. In addition, it is important to collect right and sufficient data to answer the research question (Yin, 2018). A too large sample in a qualitative study may result in less depth and loss of the uniqueness in participants experiences (Holloway & Galvin, 2017). In case studies it is more applicable to use an analytic generalization, that is to compare if the result supports earlier theories (Yin, 2018). Our case study contributes with knowledge in the field of NBI which can be compared with earlier studies and theories.

To improve the quality of the manuscript and ensure that all key areas of the study are presented the Coreq checklist has been used (Tong et al., 2007).

## Conclusion

In conclusion, NBI in a northern natural setting seems to offer a source for recovery and well-being for people with stress-related illness. Feelings of joy, reduced stress, and well-being are experienced during the programme; it also resulted in knowledge and new approaches to improve everyday life. The results show similarities with studies of NBI in other natural settings, although the intervention programme in this case study lasted for a short period. NBI may be a complement to other treatments of stress-related illness. However, further research is needed of NBI programmes lasting for longer periods, with a larger number of participants and in various northern natural settings.

## References

[cit0001] Alsén, S., Ali, L., Ekman, I., & Fors, A. (2020). Facing a blind alley - Experiences of stress-related exhaustion: A qualitative study. *BMJ Open*, 10(9), e038230. 10.1136/bmjopen-2020-038230 PMC7470632 32878762

[cit0002] Andersson, G.-E., & Marklund, R. (n.d.). Handbok för uppföljning med hjälp av ORS - outcome rating scale och SRS - session rating scale [Broschure]. (In Swedish: Handbook for follow-up using ORS – Outcome rating scale and SRS – Session rating scale). Region Västerbotten. Region V’sterbotten. https://regionvasterbotten.se/VLL/Filer/A5-Handbok-f%C3%B6r-uppf%C3%B6ljning-med-hj%C3%A4lp-av-ORS-SRS1.pdf

[cit0003] Aronsson, G., Theorell, T., Grape, T., Hammarström, A., Hogstedt, C., Marteinsdottir, I., Skoog, I., Träskman-Bendz, L., & Hall, C. (2017). A systematic review including meta-analysis of work environment and burnout symptoms. *BMC Public Health*, 17(1). 10.1186/s12889-017-4153-7 PMC5356239 28302088

[cit0004] Bengtsson, A., & Grahn, P. (2014). Outdoor environments in healthcare settings: A quality evaluation tool for use in designing healthcare gardens. *Urban Forestry & Urban Greening*, 13(4), 878–15. 10.1016/j.ufug.2014.09.007

[cit0005] Bergenheim, A., Ahlborg, G., & Bernhardsson, S. (2021). Nature-based rehabilitation for patients with long-standing stress-related mental disorders: A qualitative evidence synthesis of patients’ experiences. *International Journal of Environmental Research and Public Health*, 18(13), 6897. 10.3390/ijerph18136897 34199050 PMC8297286

[cit0006] Bielinis, E., Takayama, N., Boiko, S., Omelan, A., & Bielinis, L. (2018). The effect of winter forest bathing on psychological relaxation of young Polish adults. *Urban Forestry & Urban Greening*, 29, 276–283. 10.1016/j.ufug.2017.12.006

[cit0007] Corazon, S. S., Sidenius, U., Poulsen, D. V., Gramkow, M. C., & Stigsdotter, U. K. (2019). Psycho-physiological stress recovery in outdoor nature-based interventions: A systematic review of the past eight years of research. *International Journal of Environmental Research and Public Health*, 16(10), 1–21. 10.3390/ijerph16101711 PMC6572302 31100773

[cit0008] Cunsolo Willox, A., Harper, S. L., Ford, J. D., Landman, K., Houle, K., & Edge, V. L. (2012). “From this place and of this place:” Climate change, sense of place, and health in Nunatsiavut,Canada. *Social Science & Medicine*, 75(3), 538–547. 10.1016/j.socscimed.2012.03.043 22595069

[cit0009] Edvardsson, J. D., Sandman, P. O., & Rasmussen, B. H. (2005). Sensing an atmosphere of ease: A tentative theory of supportive care settings. *Scandinavian Journal of Caring Sciences*, 19(4), 344–353. 10.1111/j.1471-6712.2005.00356.x 16324058

[cit0010] Eriksson, T., Westerberg, Y., & Jonsson, H. (2011). Experiences of women with stress-related ill health in a therapeutic gardening program. *Canadian Journal of Occupational Therapy*, 78(5), 273–281. 10.2182/cjot.2011.78.5.2 22338294

[cit0011] Eurofound. (2018). Burnout in the workplace: A review of data and policy responses in the EU. Publications Office of the European Union. https://www.eurofound.europa.eu/publications/report/2018/burnout-in-the-workplace-a-review-of-data-and-policy-responses-in-the-eu

[cit0012] Fine, A. H., & Weaver, S. J. (2018). The human-animal bond and animal-assisted intervention. In M. van den Bosch & W. Bird (Eds.), *Oxford textbook of nature and public health: The role of nature in improving the health of a population* (pp. 132–138). Oxford University Press.

[cit0013] Flyvbjerg, B. (2006). Five misunderstandings about case-study research. *Qualitative Inquiry*, 12(2), 219–245. 10.1177/1077800405284363

[cit0014] Glise, K., Ahlborg, G., & Jonsdottir, I. H. (2014). Prevalence and course of somatic symptoms in patients with stress-related exhaustion: Does sex or age matter. *BMC Psychiatry*, 14(1). 10.1186/1471-244X-14-118 PMC3999732 24755373

[cit0015] Grahn, P. (2022). Evidensläge för behandlingsinsatser utomhus. (In Swedish: Evidence for outdoor treatment interventions.). In Å. Engström, P. Juuso, M. Liljegren, & L. L. Alfredsson (Eds.), *Vård, omsorg och rehabilitering utomhus: Teori, praktik och nya perspektiv (In Swedish: Outdoor care and rehabilitation: Theory, practice and new perspectives* (pp. 299–316). Studentlitteratur.

[cit0016] Grahn, P., Ivarsson, C., Stigsdotter, U. K., & Begtsson, I.-L. (2010). Using affordances as a health-promoting tool in a therapeutic garden. In C. W. Thompson, P. Aspinall, & S. Bell (Eds.), *Innovative approaches to researching landscape and health* (pp. 120–159). Routledge.

[cit0017] Grahn, P., Ottosson, J., & Uvnäs-Moberg, K. (2021). The oxytocinergic system as a mediator of anti-stress and instorative effects induced by nature: The calm and connection theory. *Frontiers in Psychology*, 12. 10.3389/fpsyg.2021.617814 PMC8286993 34290636

[cit0018] Grahn, P., Palsdottir, A. M., Ottosson, J., & Jonsdottir, I. H. (2017). Longer nature-based rehabilitation may contribute to a faster return to work in patients with reactions to severe stress and/or depression. *International Journal of Environmental Research and Public Health*, 14(11), 1310. 10.3390/ijerph14111310 29076997 PMC5707949

[cit0019] Grossi, G., Perski, A., Osika, W., & Savic, I. (2015). Stress-related exhaustion disorder–clinical manifestation of burnout? A review of assessment methods, sleep impairments, cognitive disturbances, and neuro-biological and physiological changes in clinical burnout. *Scandinavian Journal of Psychology*, 56(6), 626–636. 10.1111/sjop.12251 26496458

[cit0020] Håkansson, C., & Ahlborg, G. (2017). Occupations, perceived stress, and stress-related disorders among women and men in the public sector in Sweden. *Scandinavian Journal of Occupational Therapy*, 24(1), 10–17. 10.3109/11038128.2016.1170196 27141999

[cit0021] Hammell, K. W. (2004). Dimensions of meaning in the occupations of daily life. *Canadian Journal of Occupational Therapy*, 71(5), 296–305. 10.1177/000841740407100509 15633880

[cit0022] Hartig, T., van den Berg, A. E., Hagerhall, C. M., Tomalak, M., Bauer, N., Hansmann, R., Ojala, A., Syngollitou, E., Carrus, G., van Herzele, A., Bell, S., Camilleri Podesta, M. T., & Waaseth, G. (2011). Health benefits of nature experience: Psychological, social and cultural processes. In K. Nilsson, M. Sangster, C. Gallis, T. Hartig, S. de Vries, K. Seeland, & J. Schipperijn (Eds.), *Forests, trees and human health* (pp. 127–168). Springer.

[cit0023] Hasselberg, K., Jonsdottir, I. H., Ellbin, S., & Skagert, K. (2014). Self-reported stressors among patients with exhaustion disorder: An exploratory study of patient records. *BMC Psychiatry*, 14(1), 1–19. 10.1186/1471-244X-14-66 PMC3975849 24592907

[cit0024] Hofmann, S. G., Asnaani, A., Vonk, I. J. J., Sawyer, A. T., & Fang, A. (2012). The efficacy of cognitive behavioral therapy: A review of meta-analyses. *Cognitive Therapy and Research*, 36(5), 427–440. 10.1007/s10608-012-9476-1 23459093 PMC3584580

[cit0025] Holloway, I., & Galvin, K. (2017). *Qualitative research in nursing and healthcare* (4th) ed.). Wiley Blackwell.

[cit0026] Hörberg, U., Wagman, P., & Gunnarsson, A. B. (2020). Women’s lived experience of well-being in everyday life when living with a stress-related illness. *International Journal of Qualitative Studies on Health and Well-Being*, 15(1), 1–11. 10.1080/17482631.2020.1754087 PMC7191911 32320354

[cit0027] James, S. L., Abate, D., Abate, K. H., Abay, S. M., Abbafati, C., Abbasi, N., Abbastabar, H., Abd-Allah, F., Abdela, J., Abdelalim, A., Abdollahpour, I., Abdulkader, R. S., Abebe, Z., Abera, S. F., Abil, O. Z., Abraha, H. N., Abu-Raddad, L. J., Abu-Rmeileh, N. M. E., Accrombessi, M. M. K., & Murray, C. J. L. (2018). Global, regional, and national incidence, prevalence, and years lived with disability for 354 diseases and injuries for 195 countries and territories, 1990–2017: A systematic analysis for the Global Burden of Disease Study 2017. *The Lancet*, 392(10159), 1789–1858. 10.1016/S0140-6736(18)32279-7 PMC6227754 30496104

[cit0028] Johansson, G., Juuso, P., & Engström, Å. (2022). Nature-based interventions to promote health for people with stress-related illness: An integrative review. *Scandinavian Journal of Caring Sciences*. 10.1111/scs.13089 PMC9790340 35604072

[cit0029] Kaplan, S. (1995). The restorative benefits of nature: Toward an integrative framework. *Journal of Environmental Psychology*, 15(3), 169–182. 10.1016/0272-4944(95)90001-2

[cit0030] Kvale, S., & Brinkmann, S. (2014). Den kvalitativa forskningsintervjun. In *(In Swedish: The qualitative research interview)* (3rd ed.). Studentlitteratur.

[cit0031] Lindgren, B. M., Lundman, B., & Graneheim, U. H. (2020). abstraction and interpretation during the qualitative content analysis process. *International Journal of Nursing Studies*, 108, 103632. 10.1016/j.ijnurstu.2020.103632 32505813

[cit0032] Lindsäter, E., Axelsson, E., Salomonsson, S., Santoft, F., Ejeby, K., Ljótsson, B., Åkerstedt, T., Lekander, M., & Hedman-Lagerlöf, E. (2018). Internet-based cognitive behavioral therapy for chronic stress: A randomized controlled trial. *Psychotherapy and Psychosomatics*, 87(5), 296–305. 10.1159/000490742 30041167

[cit0033] Lisspers, J., Almén, N., & Sundin, R. (2014). The effects of a recovery-focused program for stress management in Women—An exploratory study. *Health*, 6(20), 2825–2836. 10.4236/health.2014.620321

[cit0034] Lundgren-Nilsson, Å., Jonsdottir, I. H., Pallant, J., & Ahlborg, G. (2012). Internal construct validity of the Shirom-Melamed burnout questionnaire (SMBQ). *BMC Public Health*, 12(1). 10.1186/1471-2458-12-1 PMC3307433 22214479

[cit0035] Miller, S. D., Duncan, B. L., Brown, J., Sparks, J. A., & Claud, D. A. (2003). The outcome rating scale: A preliminary study of the reliability, validity, and feasibility of a brief visual analog measure. *Journal of Brief Therapy*, 2(2), 91–100.

[cit0036] National Board of Health and Welfare in Sweden. (2003). *Utmattningssyndrom: Stressrelaterad psykisk ohälsa. (In Swedish: Exhaustion disorder: Stress-related mental illness)*. Bjurner och Bruno AB.

[cit0037] National Board of Health and Welfare in Sweden. (2021). ORS – Skattning av förändring. (In Swedish: ORS – Estimation of change). https://www.socialstyrelsen.se/kunskapsstod-och-regler/omraden/evidensbaserad-praktik/metodguiden/ors-skattning-av-forandring/

[cit0038] Nilmanat, K., & Kurniawan, T. (2021). The quest in case study research. *Pacific Rim International Journal of Nursing Research*, 25(1), 1–6. https://he02.tci-thaijo.org/index.php/PRIJNR/article/view/247842

[cit0039] OECD. (2012). *Sick on the job? Myths and realities about mental health and work*. https://www.oecd.org/els/mental-health-and-work-9789264124523-en.htm

[cit0040] OECD. (2013). *Mental health and work: Sweden*. https://www.oecd.org/els/emp/mental-health-and-work-sweden-9789264188730-en.htm

[cit0041] Orem, D. E. (1995). *Nursing: Concepts of practice* (5.) ed.). Mosby.

[cit0042] Palsdottir, A. M., Persson, D., Persson, B., & Grahn, P. (2014). The journey of recovery and empowerment embraced by nature - clients’ perspectives on nature-based rehabilitation in relation to the role of the natural environment. *International Journal of Environmental Research and Public Health*, 11(7), 7094–7115. 10.3390/ijerph110707094 25026080 PMC4113863

[cit0043] Pálsdóttir, A. M., Sempik, J., Bird, W., & van den Bosch, M. (2018). Using nature as a treatment option. In M. van den Bosch & W. Bird (Eds.), *Oxford textbook of nature and public health: The role of nature in improving the health of a population* (pp. 108–115). Oxford University Press.

[cit0044] Pauli, E. (2019). *Utvärdering av gröna rehab: Uppdatering av socioekonomisk analys utifrån tidigare genomförda utvärderingar. (In Swedish: Evaluation of green rehab: Update of socio-economic analysis based on previous evaluations.)*. Gothia Forum.

[cit0045] Polit, D. F., & Beck, C. T. (2021). *Nursing Research: Generating and assessing evidence for nursing practice* (Eleventh) ed.). Wolters Kluwer.

[cit0046] Salomonsson, S., Santoft, F., Lindsäter, E., Ejeby, K., Ingvar, M., Ljótsson, B., Ost, L., Lekander, M., & Hedman-Lagerlöf, E. (2020). Effects of cognitive behavioural therapy and return-to-work intervention for patients on sick leave due to stress-related disorders: Results from a randomized trial. *Scandinavian Journal of Psychology*, 61(2), 281–289. 10.1111/sjop.12590 31691305

[cit0047] Seeland, K. (2011). Landscape and health as representations of cultural diversity. In K. Nilsson, M. Sangster, C. Gallis, T. Hartig, S. de Vries, K. Seeland, & J. Schipperijn (Eds.), *Forests, trees and human health* (pp. 403–409). Springer.

[cit0048] Sempik, J., Hine, R., & Wilcox, D. (2010) Green Care: A conceptional framework (A report of the working group on health benefits of Green Care). Loughborough University. COST 866, Green Care in Agriculture. https://www.greencare.at/wp-content/uploads/sites/6/2021/01/COST-866-A-Conceptual-Framework.pdf

[cit0049] Sidenius, U., Karlsson Nyed, P., Lygum, V. L., & Stigsdotter, U. K. (2017). A diagnostic post-occupancy evaluation of the nacadia(R) therapy garden. *International Journal of Environmental Research and Public Health*, 14(8), 882. 10.3390/ijerph14080882 28783060 PMC5580586

[cit0050] Steigen, A. M., Kogstad, R., & Hummelvoll, J. K. (2016). Green care services in the Nordic countries: An integrative literature review. *European Journal of Social Work*, 19(5), 692–715. 10.1080/13691457.2015.1082983

[cit0051] Stigmar, K., Kyrö Wissler, S., Åström, M., Pálsdottir, A.-M., Grahn, P., & Petersson, I. (2016). *Naturunderstödd rehabilitering på landsbygden i region Skåne. (In Swedish: Nature-based rehabilitation in the countryside in the Skåne region)*. Sveriges Lantbruksuniversitet (In Swedish: Swedish University of Agricultural Sciences).

[cit0052] Stigsdotter, U. A., & Grahn, P. (2003). Experiencing a garden: A healing garden för people suffering from burnout diseases. *Journal of Therapeutic Horticulture*, 14, 38–49. Available from: hybridparks.eu/wp-content/uploads/downloads/2012/11/Presentation_Grahn_Lund.pdf

[cit0053] Stigsdotter, U. K., Pálsdottir, A. M., Burls, A., Chermaz, A., Ferrinen, F., & Grahn, P. (2011). Nature-based therapeutic interventions. In K. Nilsson, M. Sangster, C. Gallis, T. Hartig, S. de Vries, K. Seeland, J. Schipperijn, (Eds.), *Forests, Trees and Human Health* (pp. 127–168). Springer.

[cit0054] Swedish Social Insurance Agency. (2016). Korta analyser 2016:2 Sjukskrivning för reaktioner på svår stress ökar mest. (In Swedish: Short analyzes 2016:2 Sick leave due to reactions to severe stress increases the most.) https://www.forsakringskassan.se/wps/wcm/connect/41903408-e87d-4e5e-8f7f-90275dafe6ad/psykisk-ohalsa-korta-analyser-2016-2.pdf?MOD=AJPERES&CVID=&CACHE=NONE&CONTENTCACHE=NONE

[cit0055] Swedish Social Insurance Agency. (2020). Socialförsäkringsrapport 2020:8 sjukfrånvaro i psykiatriska diagnoser. (In Swedish: Social insurance report 2020:8 Sick leave in psychiatric diagnoses.) https://www.forsakringskassan.se/wps/wcm/connect/e12b777c-e98a-488d-998f-501e621f4714/sjukfranvaro-i-psykiatriska-diagnoser-socialforsakringsrapport-2020-8.pdf?MOD=AJPERES&CVID=

[cit0056] Tong, A., Sainsbury, P., & Craig, J. (2007). Consolidated criteria for reporting qualitative research (COREQ): A 32-item checklist for interviews and focus groups. *International Journal for Quality in Health Care: Journal of the International Society for Quality in Health Care*, 19(6), 349–357. 10.1093/intqhc/mzm042 17872937

[cit0057] van de Leur, J. C., Buhrman, M., Åhs, F., Rozental, A., & Jansen, G. B. (2020). Standardized multimodal intervention for stress-induced exhaustion disorder: An open trial in a clinical setting. *BMC Psychiatry*, 20(1), 1–14. 10.1186/s12888-020-02907-3 33153461 PMC7643309

[cit0058] van den Bosch, M., Ward Thompson, C., & Grahn, P. (2018). Preventing stress and promoting mental health. In M. van den Bosch & W. Bird (Eds.), *Oxford textbook of nature and public health: The role of nature in improving the health of a population* (pp. 108.115). Oxford University Press.

[cit0059] Wästberg, B. A., Harris, U., & Gunnarsson, A. B. (2021). Experiences of meaning in garden therapy in outpatient psychiatric care in Sweden. A narrative study. *Scandinavian Journal of Occupational Therapy*, 28(6), 415–425. 10.1080/11038128.2020.1723684 32027526

[cit0060] Wiegner, L., Hange, D., Björkelund, C., & Ahlborg, G. (2015). Prevalence of perceived stress and associations to symptoms of exhaustion, depression and anxiety in a working age population seeking primary care - an observational study. *BMC Family Practice*, 16(1), 1–8. 10.1186/s12875-015-0252-7 25880219 PMC4377029

[cit0061] Yin, R. K. (2018). *Case study research and applications: Design and methods* (6th) ed.). SAGE.

[cit0062] Zulkosky, K. (2009). Self-efficacy: a concept analysis. *Nursing Forum*, 44(2), 93–102. 10.1111/j.1744-6198.2009.00132.x

